# Quantitative biparametric analysis of hybrid ^18^F-FET PET/MR-neuroimaging for differentiation between treatment response and recurrent glioma

**DOI:** 10.1038/s41598-019-50182-4

**Published:** 2019-10-10

**Authors:** Johannes Lohmeier, Georg Bohner, Eberhard Siebert, Winfried Brenner, Bernd Hamm, Marcus R. Makowski

**Affiliations:** 1 Charité Universitätsmedizin Berlin, Department of Radiology, Campus Charité Mitte (CCM), Charitéplatz 1, 10117 Berlin, Germany; 2 Charité Universitätsmedizin Berlin, Department of Neuroradiology, Campus Charité Mitte (CCM), Charitéplatz 1, 10117 Berlin, Germany; 3 Charité Universitätsmedizin Berlin, Department of Nuclear Medicine, Campus Virchow-Klinikum (CVK), Augustenburger Platz 1, 13353 Berlin, Germany

**Keywords:** CNS cancer, Cancer imaging

## Abstract

We investigated the diagnostic potential of simultaneous ^18^F-FET PET/MR-imaging for differentiation between recurrent glioma and post-treatment related effects (PTRE) using quantitative volumetric (3D-VOI) lesion analysis. In this retrospective study, a total of 42 patients including 32 patients with histologically proven glioma relapse and 10 patients with PTRE (histopathologic follow-up, n = 4, serial imaging follow-up, n = 6) were evaluated regarding recurrence. PET/MR-imaging was semi-automatically analysed based on FET tracer uptake using conservative SUV thresholding (isocontour 80%) with emphasis on the metabolically most active regions. Mean (relative) apparent diffusion coefficient (ADCmean, rADCmean), standardised-uptake-value (SUV) including target-to-background (TBR) ratio were determined. Glioma relapse presented higher ADCmean (MD ± SE, 284 ± 91, p = 0.003) and TBRmax (MD ± SE, 1.10 ± 0.45, p = 0.02) values than treatment-related changes. Both ADCmean (AUC ± SE = 0.82 ± 0.07, p-value < 0.001) and TBRmax (AUC ± SE = 0.81 ± 0.08, p-value < 0.001) achieved reliable diagnostic performance in differentiating glioma recurrence from PTRE. Bivariate analysis based on a combination of ADCmean and TBRmax demonstrated highest diagnostic accuracy (AUC ± SE = 0.90 ± 0.05, p-value < 0.001), improving clinical (false negative and false positive) classification. In conclusion, biparametric analysis using DWI and FET PET, both providing distinct information regarding the underlying pathophysiology, presented best diagnostic accuracy and clinical benefit in differentiating recurrent glioma from treatment-related changes.

## Introduction

Glioma recurrence after multimodal treatment with surgery and/or radio-/chemotherapy is still common. Early detection of relapse and immediate therapy play an important role in clinical follow-up. Neurosurgical biopsy is considered the current diagnostic standard, but it involves the hazards of invasive surgical intervention, such as neurological deficits, post-operative infection or intracranial haematoma. Thus, there is great interest in the development and application of non-invasive diagnostic approaches, potentially reducing the risk of severe side effects for patients as well as health care costs. Differentiation between recurrent glioma and post-treatment related effects (PTRE), such as pseudoprogression and radionecrosis, can be challenging as both share clinical symptoms and morphological imaging characteristics^1,2^. Therefore, as a clinical guideline, the Response Assessment in Neuro-Oncology (RANO) Working Group published criteria for response evaluation^3,4^, taking more advanced imaging approaches into consideration, such as perfusion magnetic resonance imaging (pMRI), magnetic resonance spectroscopy (MRS), single-photon emission computed tomography (SPECT) or positron emission tomography (PET). Commonly, however, many of these alternative methods are not established in clinical routine due to their limited clinical availability and considerable complexity. In particular, the ^18^F-labelled non-natural amino acid ^18^F-fluoroethyl-L-tyrosine (FET) emerged as a promising PET tracer candidate, targeting the tumour metabolism without relevant background signal, favourable half-life and efficient routine tracer production^5–7^. In contrast to long-established tracers in the field of neurooncology, such as ^18^F-fludeoxyglucose (FDG) and ^11^C-L-methionine (MET), FET was reported to exhibit relatively low uptake in inflammation^8,9^. In the last few years, several studies provided evidence that FET is a useful tracer for diagnosis of primary brain tumours, treatment planning and tumour grading^7^.

In clinical routine, PET tracer uptake evaluation is either performed using qualitative visual assessment, which is subjective and dependent on clinical experience, or using semi-quantitative approaches based on standardised-uptake-value (SUV). Upon tumour segmentation using a 2D region-of-interest (2D-ROI), which is more common, or a 3D volume-of-interest (3D-VOI), which determines tracer uptake in a more robust manner^10^, quantitative image-derived parameters can be computed from the respective delineation.

Prior to the adoption of hybrid PET/MR-imaging, sequential image acquisition with alignment during post-processing was standard practice. However, with the recent development of hybrid PET/MRI, simultaneous multimodal imaging was introduced, which combines the informative value of functional and structural MR-imaging as well as metabolic PET-imaging with the benefit of high spatial and temporal conformance and improved technical correction methods^11^.

In the current study, we investigated whether differentiation between recurrent glioma and treatment-related changes could be improved by simultaneous PET/MR-imaging, including diffusion weighted imaging (DWI) and FET PET, using quantitative volumetric (3D-VOI) lesion analysis with emphasis on the metabolically most active regions.

## Patients and Methods

### Patient collective

**Figure 1:**
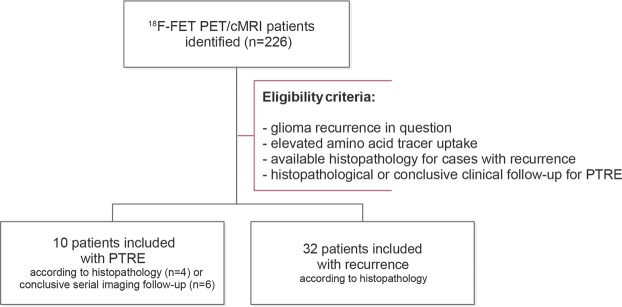
Flow diagram illustrating the study selection process. Between 2017 and 2018, we identified 226 patients, who received hybrid ^18^F-FET PET/cMRI. According to the eligibility criteria, a total of 42 patients with glioma recurrence in question were included into our retrospective study.

Approval from the institutional ethics board (Charité University Hospital Berlin, Germany, EA1/175/18) was obtained. The study was performed in accordance with the principles of the Helsinki Declaration. In this retrospective study, a total of 42 consecutive patients (PTRE/recurrence, 10/32, female/male, 18/24, age (M ± SD), 47 ± 13 a, high-/low-grade glioma (HGG/LGG), 40/2, O6-methylguanine DNA methyltransferase (MGMT+/−) mutation, 22/14 (6 unknown), isocitrate dehydrogenase 1 (IDH1+/−) mutation, 21/18 (3 unknown), loss of heterozygosity on chromosomes 1p and 19q (LOH1p/19q+/−), 10/17 (15 unknown)) with hybrid FET PET/MR-imaging (Charité University Hospital Berlin, Germany, 2017–2018) were included, as shown in Fig. 1. According to the eligibility criteria, all patients with recurrent glioma presented available histopathology, while patients with PTRE either had follow-up with histopathology (n = 4) or conclusive serial imaging (n = 6, ≥ 4 MRI follow-up examinations) in accordance to the Response Assessment in Neuro-Oncology (RANO) criteria and the conclusion of an interdisciplinary board of clinical experts. Conventional clinical evaluation was derived from the final conclusion of the written imaging report. All patients gave written informed consent regarding the use of their data.

### PET-MR Imaging protocols

Simultaneous FET PET/MRI acquisition was performed on MAGNETOM Biograph mMR (Siemens Healthcare, Erlangen, Germany; 3 T PET/MRI hybrid, 45 mT/m maximum gradient amplitude, 200 T/m/s maximum gradient slew rate, LSO crystal, 4.3 mm average spatial resolution at FWHM at 1 cm, 15.0 kcps/MBq sensitivity at center, 13.8 kcps/MBq sensitivity at radial offset of 10 cm^12^). After intravenous FET tracer injection (M ± SD, 163 ± 23 MBq; 180 MBq standard-dose for adults with body-weight ≥ 60 kg, individual dose is calculated according to a weight scale for body-weight < 60 kg) simultaneous FET PET/MRI acquisition was performed in list-mode for up to 60 minutes. Gadolinium (Gd) contrast administration was applied according to the patient’s total body-weight.

PET/MRI examinations included, inter alia, a transversal T1-weighted ultrashort echo time (UTE) sequence for attenuation and scatter correction (TR/TE1/TE2 = 4.64/0.07/2.46 ms; flip-angle = 10°; matrix-size = 192 × 192 × 192; voxel-size = 1.6 × 1.6 × 1.6 mm), PET acquisition, DWI/ep2d-DIFF 3-scan trace (TR/TE = 10000/101 ms; matrix-size = 230 × 230 × 230; voxel-size = 1.2 × 1.2 × 3.0 mm; thickness = 3 mm; slices = 48; apparent diffusion coefficient (ADC) map; diffusion-scheme = bipolar; diffusion-weighting = 2; b-values = 0 s/mm^2^, 1000 s/mm^2^; echo-spacing = 1.08 ms; EPI-factor = 192; bandwidth = 1002 Hz/Px) and post-contrast T1/FL2D-TRA (TR/TE = 250/2.66 ms; flip-angle = 70°; matrix-size = 230 × 230 × 230; voxel-size = 0.7 × 0.7 × 5.0 mm; thickness = 5 mm; slices = 30). PET was sorted into a sinogram, which was reconstructed into transaxial slices using an iterative ordered-subset expectation maximisation algorithm (OSEM) with 3 iterations and 21 subsets (matrix-size = 344 × 344 × 127; voxel-size = 1.0 × 1.0 × 2.3 mm; zoom = 2; gaussian-filter = 3 mm). Emission data were corrected for decay, randoms, dead time, scatter and attenuation.

### Volumetric image analysis

Image analysis was performed using OsiriX MD 10. Attenuation corrected PET was matched to the respective apparent diffusion coefficient (ADC) map. Using conservative SUV thresholding (isocontour 80%, IC80; threshold determined in a pilot experiment) with emphasis on the metabolically most active regions, a volume-of-interest (3D-VOI) was semi-automatically delineated based on attenuation-corrected PET. Mean background signal was computed from a contralateral 2D-ROI with similar size (unaffected brain tissue including grey and white matter) on a representative slice (slice with highest mean uptake within the 3D-VOI), as an adaptation of recommendations from the German guideline^13^. Where more than one lesion was apparent, the most prominent lesion (greatest lesion size, contrast-enhancement and PET tracer uptake, closest to the resection cavity or primary tumour location) was analysed.

Apparent diffusion coefficients (all reported values in 10^−6^ mm^2^/s) were determined using the same 3D-VOI based on high tracer uptake upon marginal adaptation to avoid overlap with resection cavity or cerebrospinal fluid space. Relative apparent diffusion coefficient (rADC) and target-to-background (TBR) ratio were calculated from standardised-uptake-value (SUVmean, SUVmax) or apparent diffusion coefficient (ADCmean) and the unaffected contralateral background signal (ratio of lesion signal and contralateral mean background signal).

### Statistical analysis

Receiver Operating Characteristic (ROC) curves were computed with respective Area Under the Curve (AUC), p-value, sensitivity and specificity. Youden’s index was determined to find the best cut-off point (independent from prevalence). ROC curves from univariate and bivariate analysis were compared for differences using a non-parametric approach. Biparametric analysis was performed in a step-wise approach (each parameter was evaluated separately, one parameter above threshold was classified as recurrence) using a binary model. Comparative analysis between recurrence (REC) and post-treatment-related (PTRE) groups were performed using independent samples t-tests (reported values show mean and standard error). p-value < 0.05 was considered statistically significant.

## Results

### Evaluation of DWI- and FET PET-derived imaging parameters

**Figure 2:**
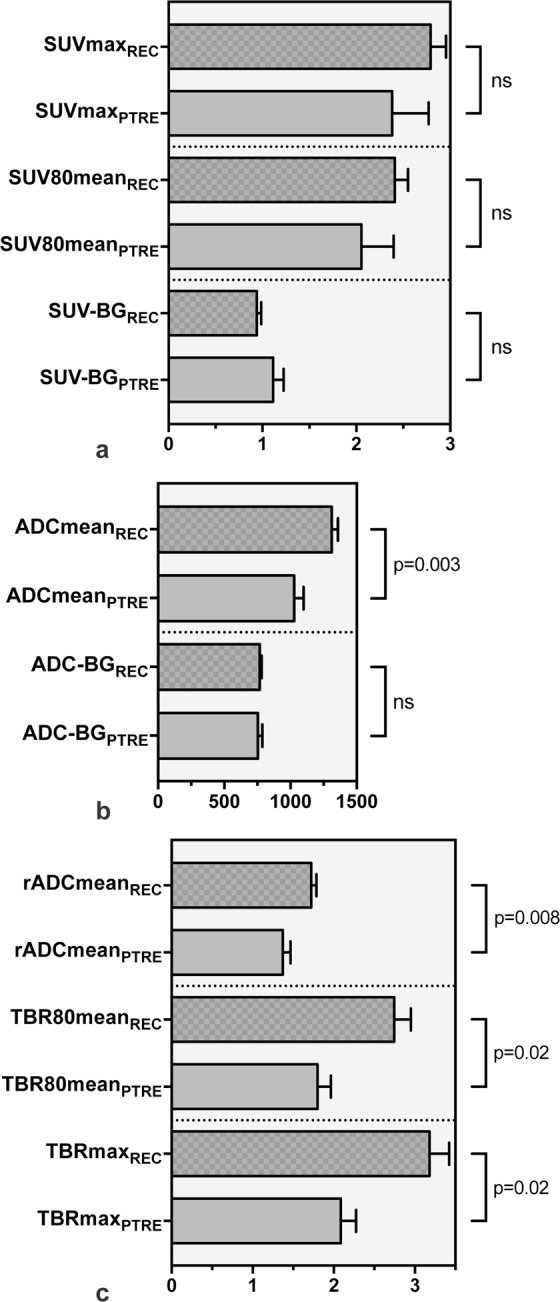
Bar graph diagrams comparing quantitative imaging parameters from FET PET and DWI between recurrent glioma (REC) and treatment-related effects (PTRE) groups. First panel (a) shows FET PET parameters (SUVmax, SUV80mean and SUV-BG). Second panel (b) demonstrates DWI parameters (ADCmean, ADC-BG) showing a statistically significant difference between recurrence and treatment-related changes with regard to ADCmean (p = 0.003). No significant differences between background values were apparent. Last panel shows relative imaging parameters for both modalities (rADCmean, TBRmax and TBR80mean). Statistically significant differences for rADCmean (p = 0.008), TBR80mean (p = 0.02) and TBRmax (p = 0.02) were found. Independent samples t-test, p-value < 0.05 was considered statistically significant. M ± SE.

Recurrent glioma presented higher ADCmean (1313 (REC) vs. 1029 (PTRE), MD ± SE, 284 ± 91, p = 0.003), rADCmean (1.72 (REC) vs. 1.37 (PTRE), MD ± SE, 0.349 ± 0.125, p = 0.008), TBR80mean (2.75 (REC) vs. 1.80 (PTRE), MD ± SE, 0.95 ± 0.38, p = 0.02) and TBRmax (3.18 (REC) vs. 2.09 (PTRE), MD ± SE, 1.10 ± 0.45, p = 0.02) values than post-treatment related effects, as shown in Fig. 2.

Due to conservative SUV thresholding with emphasis on regions with highest metabolic activity, mean and maximum SUV demonstrated high positive correlation for both recurrent glioma (R = 0.999, 95%-CI = 0.997–0.999, p < 0.001) and post-treatment related effects (R = 0.999, 95%-CI = 0.999–1.000, p < 0.001) groups.

No statistically significant association was apparent for SUV (0.94 (REC) vs. 1.11 (PTRE), MD ± SE, −0.174 ± 0.107) or ADC (768 (REC) vs. 755 (PTRE), MD ± SE, 13 ± 29) contralateral background signals (see Fig. 2).

### Diagnostic performance of DWI, FET PET and biparametric analysis

**Figure 3:**
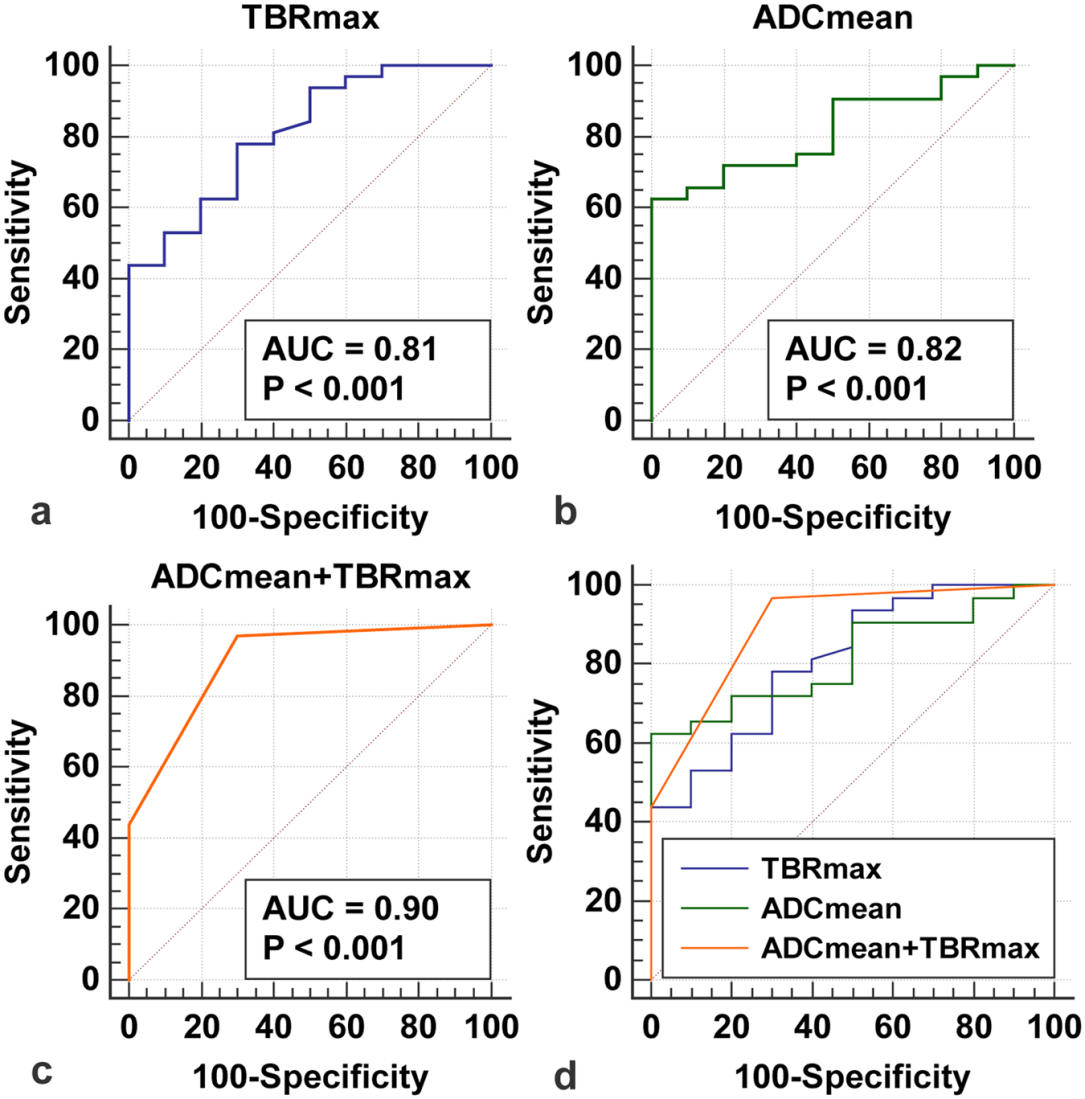
Receiver Operating Characteristic (ROC) analysis. ROC curves for TBRmax (**a**), ADCmean (**b**) and biparametric analysis of DWI- and FET PET-derived parameters (c, ADCmean and TBRmax) were illustrated. Biparametric analysis (**c**) presented highest AUC (Area Under the Curve). Last panel (d) shows a comparison between ROC curves. p-value < 0.05 was considered statistically significant.

Mean apparent diffusion coefficient (AUC ± SE, 0.82 ± 0.07, p-value < 0.001, 95%-CI = 0.666–0.918) presented good diagnostic performance with an optimal cut-off at ADCmean > 1254 (62% sensitivity, 100% specificity), as shown in Fig. 3b and Table 1.

**Table 1:** Diagnostic measures of clinical assessment and quantitative PET/MRI analysis.

	Sensitivity/Specificity	Positive/negative predictive value	AUC
**ADCmean + TBRmax**	**97%/60%**	**71%/95%**	**0.90**
**ADCmean > 1254**	62%/100%	100%/72%	0.82
**TBRmax > 2**	81%/60%	67%/76%	0.81
**Clinical rating**	91%/44%	62%/83%	—

Biparametric analysis using DWI- and FET PET-derived parameters presents improved diagnostic measures compared to clinical assessment.

Maximum target-to-background ratio (AUC ± SE = 0.81 ± 0.08, p-value < 0.001, 95%-CI = 0.660–0.915) showed similar diagnostic performance (see Fig. 3a and Table 1) with 81% sensitivity and 60% specificity at the clinically established threshold TBRmax > 2.

**Figure 4:**
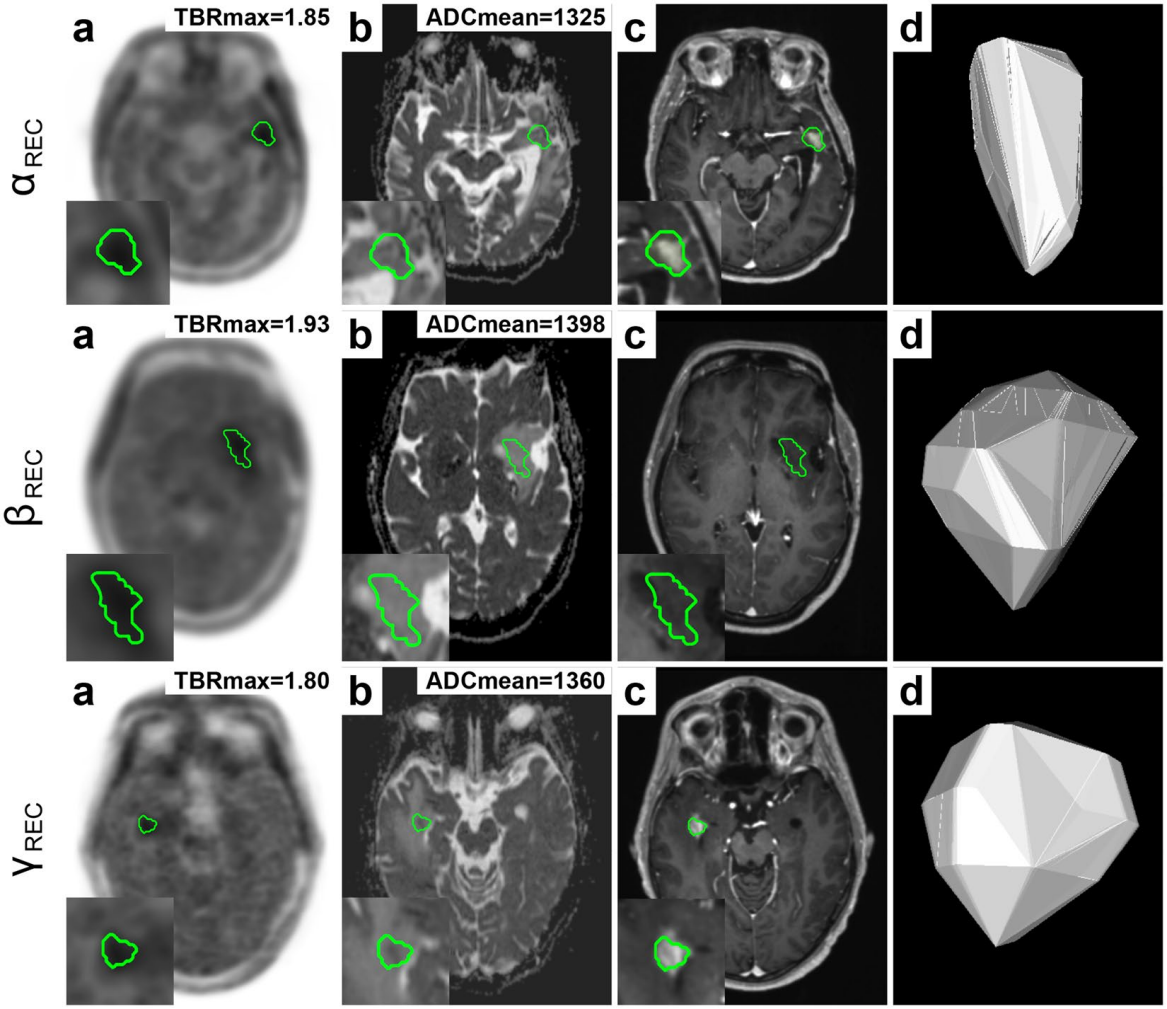
Additional value of diffusion weighted imaging (DWI) for differentiation between recurrent glioma (REC) and treatment-related effects (PTRE). Semi-automated delineation (IC80) of lesion VOI based on PET (**a**) was transferred to the respective ADC map (**b**). The following panels show post-contrast T1W (**c**) and a 3D lesion model (**d**) for illustration. Each row (α, β, γ) shows a patient with recurrent glioma. While TBRmax was at the border of the clinically established threshold (TBRmax > 2), ADCmean values were above threshold (ADCmean > 1254) suggesting recurrence.

**Figure 5:**
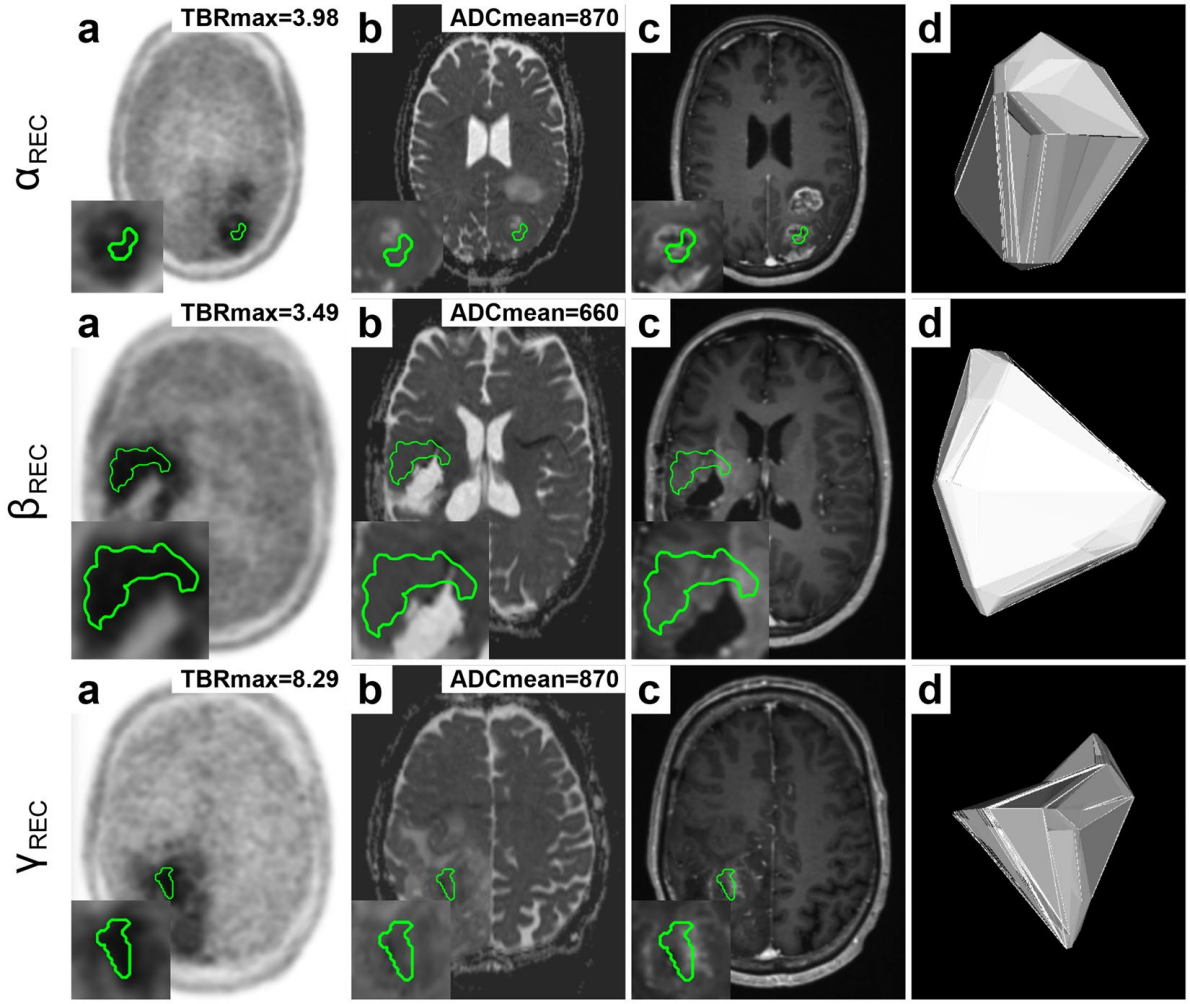
Glioma recurrence with restricted diffusion. Semi-automated delineation (IC80) of lesion VOI based on PET (**a**) was transferred to the respective ADC map (**b**). The following panels show post-contrast T1W (**c**) and a 3D lesion model (**d**) for illustration. Each row (α, β, γ) shows a patient with recurrence. While TBRmax is above the clinically established threshold (TBRmax > 2) suggesting recurrence, ADCmean shows lower readings (ADCmean < 900) indicating diffusion restriction due to high tumour cellularity.

ADCmean presented additional diagnostic value, when TBRmax was close to the clinically established threshold (TBRmax > 2), effectively improving clinical detection and decision-making, as demonstrated in Fig. 4. Few cases showed recurrent glioma, as suggested by increased TBRmax > 2, with considerably lower mean apparent diffusion coefficient (ADCmean < 900), suggesting diffusion restriction due to high tumour cellularity (see Fig. 5).

When both DWI- and FET-derived parameters were combined in biparametric approach, the highest accuracy (AUC ± SE = 0.90 ± 05, p-value < 0.001, 95%-CI = 0.768–0.971) was achieved (see Fig. 3c). Compared to conventional clinical assessment, as shown in Table 1, biparametric analysis with ADCmean > 1254 and TBRmax > 2 presented increased sensitivity (97% vs. 91%) and specificity (60% vs. 44%), improving clinical false negative (−6%) and false positive (−16%) classification. However, no statistically significant differences regarding diagnostic power were observed between bivariate analysis and univariate analysis with either TBRmax (difference in AUC ± SE = 0.09 ± 0.05, 95%-CI = −0.00702–0.185, p = 0.07) or ADCmean (difference in AUC ± SE = 0.08 ± 0.05; 95%-CI = −0.0197–0.188, p = 0.11).

**Figure 6:**
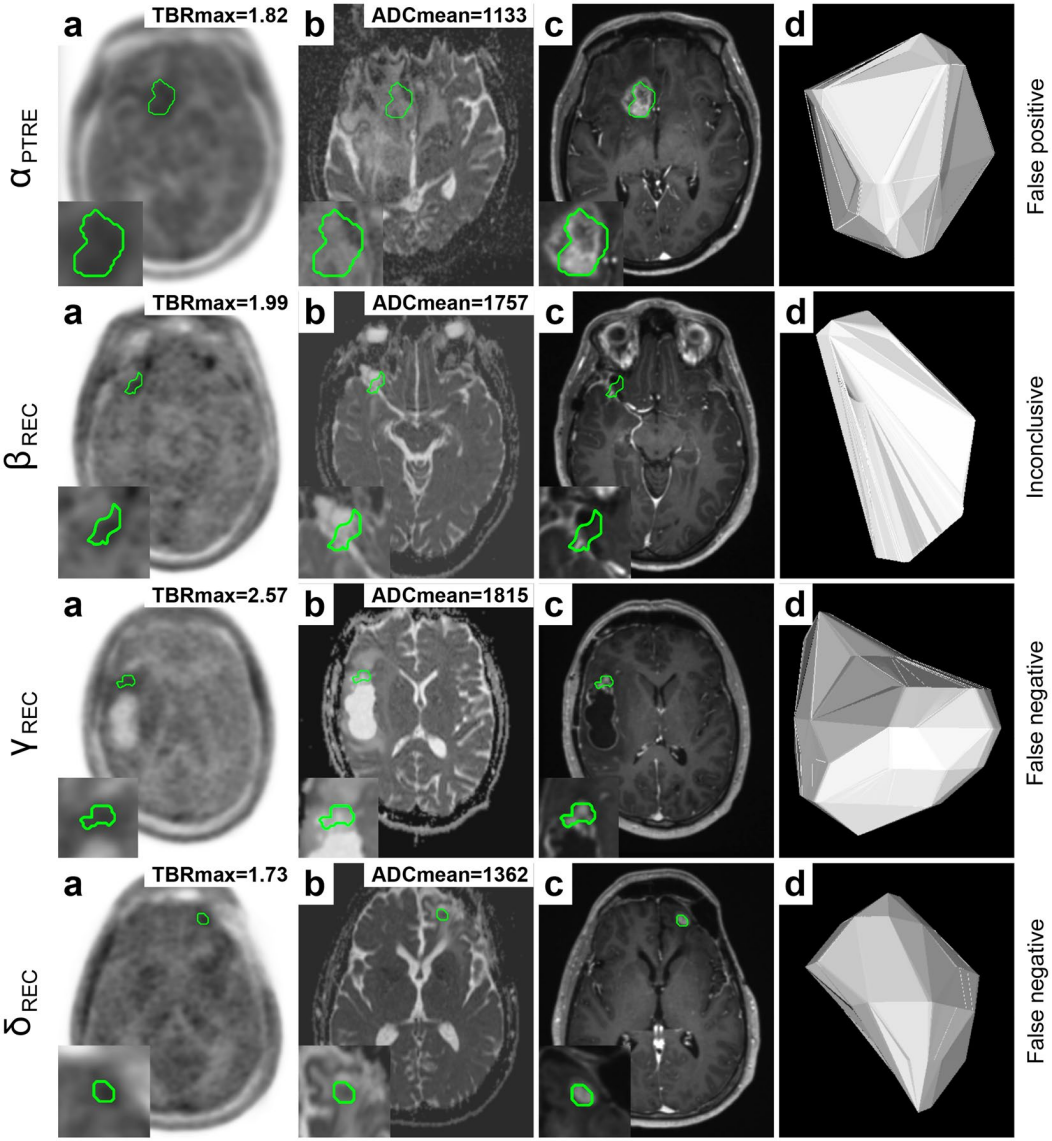
Benefit of biparametric analysis of DWI- and FET PET-derived parameters compared to conventional clinical evaluation. Semi-automated delineation (IC80) of lesion VOI based on PET (**a**) was transferred to the respective ADC map (**b**). The following panels show post-contrast T1W (**c**) and a 3D lesion model (**d**) for illustration. Labels on the most right show conventional clinical assessment. First row (α) shows a patient with PTRE, which was originally classified as glioma recurrence, which led to surgical intervention. Second row (β) shows a case with an inconclusive clinical evaluation, which was later shown to be glioma relapse. The following rows (γ, δ) present patients with relapse according to histopathology, originally classified as treatment-related changes. Clinical assessment would have benefitted from biparametric analysis using TBRmax (TBRmax > 2) and ADCmean (ADCmean > 1254), reducing clinical false positive and negative classification.

Benefit of biparametric analysis compared to conventional clinical assessment is demonstrated in Fig. 6, indicating that either surgery could have been prevented or earlier detection could have been achieved. First row (see Fig. 6, panel α) demonstrates a patient with a treated WHO III° oligodendroglioma (MGMT+, IDH1+, LOH1p/19q+), originally classified as relapse. In concordance with histopathology, TBRmax (TBRmax = 1.82) and ADCmean (ADCmean = 1133) indicated treatment-related changes. Second row (see Fig. 6, panel β) presents a patient with a WHO III° astrocytoma (MGMT+, IDH1+, LOH1p/19q−) with an ambiguous report concluding that no high-grade recurrence was detected. An MRI follow-up examination (after 4 months) gave rise to the suspicion of relapse, which was later histologically proven (5 months after hybrid PET/MRI). While TBRmax was close to the threshold (TBRmax = 1.99), ADCmean (ADCmean = 1757) suggested recurrent glioma. Third row (see Fig. 6, panel γ) shows recurrence of a WHO III° oligodendroglioma (MGMT−, IDH1−), originally categorised as treatment-related effect. Both TBRmax (TBRmax = 2.57) and ADCmean (ADCmean = 1815) suggested recurrence. A follow-up examination (after 3 months) suggested tumour progression (with midline shift), which was later histologically proven in surgery (4 months after hybrid PET/MRI). Fourth row (see Fig. 6, panel δ) presents a patient with WHO III° oligodendroglioma (MGMT−, IDH1+, LOH1p/19q+), which was classified as post-treatment related effect. While TBRmax was below the clinically established threshold (TBRmax = 1.73), ADCmean (ADCmean = 1362) indicated recurrence. Follow-up MRI examinations (after 3.5 and 6 months) suggested relapse with progressive contrast enhancement, which eventually led to surgery (7 months after hybrid PET/MRI) confirming recurrent glioma.

## Discussion

In the current study, we demonstrated that the mean apparent diffusion coefficient (ADCmean) in the metabolically most active regions was higher in patients with recurrent glioma than in patients with post-treatment related effects. Both DWI and FET PET facilitated reliable differentiation between glioma recurrence and post-treatment related effects. Furthermore, we showed that mean apparent diffusion coefficient (ADCmean) was valuable, where maximum target-to-background (TBRmax) ratio remained inconclusive. Thus, highest accuracy (90%) was achieved when both DWI- and ^18^F-FET-derived parameters were combined in a biparametric approach, which was superior to evaluating maximum target-to-background (TBRmax) ratio or mean apparent diffusion coefficient (ADCmean) alone. When compared to conventional clinical assessment, both false negative (−6%) and false positive (−16%) classification rates were improved by biparametric analysis, which results in earlier detection of recurrence or avoidance of surgery.

While reports regarding the application of quantitative DWI-derived parameters for the differentiation between recurrence and treatment-related effects in glioma patients are inconsistent^2,7^, our results showed that glioma relapse presents higher mean apparent diffusion coefficient (ADCmean) compared to treatment-related effects, presumably due to tumour necrosis in the centre of solid tumours. While this finding is in accordance with some earlier reports^14,15^, a larger share of previous studies reported that treatment-related effects show higher apparent diffusion coefficient (ADC) values than recurrent glioma^2^.

In these studies, the presumed mechanism for lower ADC values in glioma relapse is higher cellularity due to rapid cell proliferation, resulting in relatively impaired diffusivity. This observation is in accordance with individual cases in our study, showing recurrent glioma with lower ADC values (ADCmean < 900), likely reflecting high tumour cellularity in regions with high tracer uptake, which is in contrast to the larger share of patients in this study, presenting increased ADC in recurrent glioma. In a study by Berro *et al*.^16^, no link was found between increased ^18^F-fluoro-L-thymidine (FLT) uptake as a marker for cellular proliferation and decreased ADC in preoperative glioblastoma multiforme (GBM). 

The current study expands knowledge on tumour metabolism and concurrent diffusivity, showing that the metabolically most active tumour sites, where glucose and amino acid uptake are known to be deregulated^17^, present increased ADC values. We assume that the underlying mechanism for increased diffusivity in regions with high metabolic activity is tumour necrosis in close proximity to areas with high growth rates, where tumour cells eventually become hypoxic and glucose-depleted due to insufficient oxygen and nutrient supply, resulting in tumour cell death^17^. A similar finding was reported by Gadda *et al*.^14^, which determined increased ADC values for high-grade glioma (HGG) in areas with lactate and lipid compounds as markers for necrosis and hypoxia using magnetic resonance spectroscopy (MRS) and conventional MRI. Discrepancies observed between studies are likely attributable to methodological and technical differences (e.g. manual lesion segmentation, delineation based on contrast-enhancement, perilesional measurements, or less representative 2D-ROIs) in contrast to the current study’s rationale of measuring ADC values exclusively in regions with highest tracer uptake.

While mean apparent diffusion coefficient (ADCmean) was elevated in most patients with recurrent glioma, few individual cases showed high diffusion restriction, therefore, suggesting that recurrence should also be considered at lower levels (ADCmean < 900) in regions with particularly high metabolic activity (TBRmax > 2). Moreover, our findings suggest that there is no direct benefit in computing the relative apparent diffusion coefficient (rADCmean) in contrast to the clinically established target-to-background (TBR) ratio for FET PET, which provided additional informative value.

An earlier study from Jena *et al*.^18^ indicated that multiparametric imaging using a combination of FET PET, dynamic susceptibility contrast (DSC) and magnetic resonance spectroscopy (MRS) may achieve a maximum AUC of 0.94 based on logistic regression (n = 26, PTRE/recurrence, 7/19, histopathological/imaging follow-up, 9/17). However, lesions were manually delineated based on contrast enhancement and/or FET tracer uptake and no practical implementation of multiparametric assessment was provided.

To date, several methodological approaches for the discrimination between glioma relapse and treatment-related changes were proposed, such as the evaluation of structural criteria^2^, qualitative assessment of T1-/T2-mismatch^19^, defined as the contrast-enhanced lesion on a T1W image without matching lesion in a T2W image, or quantitative evaluation of the lesion quotient (LQ)^20^, which is the ratio of lesion size in a T2W image and its corresponding size on a T1W image. However, in previous studies, some of these qualitative and semi-quantitative methods were shown to yield inconsistent results^21,22^.

In recent years, it was shown that FET PET is a valuable imaging method for the diagnosis and staging of primary brain tumours and pre-therapeutic planning^7^. The current study demonstrated that simultaneous biparametric FET PET/MR-imaging is a useful diagnostic approach for the differentiation between recurrent glioma and treatment-related changes, which benefits from the integration and combination of structural, metabolic, and functional imaging as well as the high spatial and temporal conformance.

Limitations of the current study are a heterogeneous patient collective, restricted sample size, retrospective study design as well as semi-automated lesion segmentation. A comparison of the current study’s results to the existing literature is limited due to the differences in methodology, as discussed above. Moreover, it should be taken into account that our findings might not apply to low malignant and slow-growing brain tumours, considering the predominance of high-grade glioma in this study. In contrast to manual delineation, which is subject to intra- and inter-rater variation, semi-automated segmentation using isocontours, where thresholds commonly vary between 40–90%^23,24^, presents a more objective and robust approach, despite not being (entirely) operator-independent. With regard to the mean apparent diffusion coefficient (ADCmean), where the threshold for differentiation was determined from the current patient collective, optimistic performance estimates are possible. Therefore, future studies should investigate and validate simultaneous bi- and multiparametric PET/MR-imaging in prospective multicentric trials. Further research should explore the potential of fully automated feature extraction and radiomics for identification of novel imaging biomarkers as well as radiogenomics for the exploration of correlations between imaging phenotypes and genomics.

In conclusion, biparametric analysis using DWI and FET PET in hybrid PET/MR-imaging, both providing distinct information regarding the underlying physiology, presented best diagnostic accuracy and clinical benefit in differentiating recurrent glioma from treatment-related effects.

## Data Availability

Authors confirm that all relevant data are included in the article.
