# Author Correction: Quantitative biparametric analysis of hybrid ^18^F-FET PET/MR-neuroimaging for differentiation between treatment response and recurrent glioma

**DOI:** 10.1038/s41598-020-63858-z

**Published:** 2020-04-22

**Authors:** Johannes Lohmeier, Georg Bohner, Eberhard Siebert, Winfried Brenner, Bernd Hamm, Marcus R. Makowski

**Affiliations:** 1 Charité Universitätsmedizin Berlin, Department of Radiology, Campus Charité Mitte (CCM), Charitéplatz 1, 10117 Berlin, Germany; 2 Charité Universitätsmedizin Berlin, Department of Neuroradiology, Campus Charité Mitte (CCM), Charitéplatz 1, 10117 Berlin, Germany; 3 Charité Universitätsmedizin Berlin, Department of Nuclear Medicine, Campus Virchow-Klinikum (CVK), Augustenburger Platz 1, 13353 Berlin, Germany

Correction to: *Scientific Reports* 10.1038/s41598-019-50182-4, published online 10 October 2019

This Article contains errors.

In the Discussion section,

“Thus, highest accuracy (90%) was achieved when both DWI- and ^18^F-FET-derived parameters were combined in a biparametric approach, which was superior to evaluating maximum target-to-background (TBRmax) ratio or mean apparent diffusion coefficient (ADCmean) alone.”

should read:

“Thus, highest accuracy (88%) was achieved when both DWI- and ^18^F-FET-derived parameters were combined in a biparametric approach, which was superior to evaluating maximum target-to-background (TBRmax) ratio or mean apparent diffusion coefficient (ADCmean) alone.”

In the legend of Figure 3,

“Receiver Operating Characteristic (ROC) analysis. ROC curves for TBRmax (**a**), ADCmean (**b**) and biparametric analysis of DWI- and FET PET-derived parameters (c, ADCmean and TBRmax) were illustrated. Biparametric analysis (**c**) presented highest AUC (Area Under the Curve). Last panel (**d**) shows a comparison between ROC curves. p-value < 0.05 was considered statistically significant.”

should read:

“Receiver Operating Characteristic (ROC) analysis. ROC curves for TBRmax (**a**), ADCmean (**b**) and biparametric analysis of DWI- and FET PET-derived parameters (c, ADCmean and TBRmax) were illustrated. Biparametric analysis (**c**) using optimal criterions based on Youden index (TBRmax > 2.1196 and ADCmean > 1253.76) presented highest AUC (Area Under the Curve). Last panel (**d**) shows a comparison between ROC curves. p-value < 0.05 was considered statistically significant.”

Additionally, in Table 1 the values in the column entitled ‘Positive/Negative predictive value’ are incorrect, and the column entitled ‘Positive/Negative Likelihood Ratio’ was omitted.

The correct Table 1 appears below.

**Table 1:** Diagnostic measures of clinical assessment and quantitative PET/MRI analysis.

	Sensitivity/pecificity	Positive/Negative predictive value	Positive/Negative Likelihood Ratio	AUC
**ADCmean + TBRmax**	**97%/60%**	**89%/86%**	**2.42/0.05**	**0.90**
**ADCmean > 1254**	62%/100%	100%/45%	− /0.38	0.82
**TBRmax > 2**	81%/60%	87%/50%	2.03/0.31	0.81
**Clinical rating**	91%/44%	85%/57%	1.63/0.21	−

Biparametric analysis using DWI- and FET PET-derived parameters presents improved diagnostic measures compared to clinical assessment.

